# O-Arm-Navigated, Robot-Assisted Versus Conventional CT Guided Radiofrequency Ablation in Treatment of Osteoid Osteoma: A Retrospective Cohort Study

**DOI:** 10.3389/fsurg.2022.881852

**Published:** 2022-05-02

**Authors:** Tian-Long Wang, Yi-Ping Luo, Zi-Fei Zhou, Jun-Feng Liu, Xiao-Dong Hou, Shao-Hua Jia, Long-Po Zheng

**Affiliations:** Department of Orthopedics, Shanghai Tenth People's Hospital, School of Medicine, Tongji University, Shanghai, China

**Keywords:** osteoma, osteoid, imaging, three-dimensional, robotic surgical procedures, radiofrequency ablation, minimally invasion

## Abstract

**Background:**

Osteoid osteoma is a common benign bone tumor, and clinically there is severe local pain that typically worsens at night. The conventional CT-guided radiofrequency ablation (RFA) was widely used in the treatment of osteoid osteoma (OO), which could result in some radiation-related and imprecise complications due to the overdose of radiation exposure. This study aimed to compare the surgical effect of robot-assisted RFA with O-arm navigation and conventional CT-guided RFA in the treatment of OO.

**Methods:**

Sixty-two patients who underwent robot-assisted RFA with O-arm navigation (Robot-RFA, *n* = 24) or CT-guided RFA (CT-RFA, *n* = 38) were included in this retrospective cohort study. The mean follow-up time was 23.3 months. The intra-operative data, primary technical success rate, visual analog scale (VAS), and post-operative complications were analyzed.

**Results:**

Primary technical success was obtained in 23 patients who had robot-assisted RFA, and 35 patients who had conventional CT-guided RFA. One patient in Robot-RFA group and three patients in CT-RFA group with pain recurrence received repeat-RFA and had a secondary success. Mean operation time and dose of radiation exposure were lower in Robot-RFA group than that in CT-RFA group. The Robot-RFA group took fewer K-wire adjustment times for each patient than the CT-RFA group. There was a statistically significant difference in the mean operation time, dose of radiation exposure, and K-wire adjustment times between the groups (*p* < 0.05). No complications associated with the procedure were reported in the two groups during the follow-up period.

**Conclusion:**

Robot-assisted RFA with O-arm navigation is a safer and more precise strategy in the treatment of osteoid osteoma with less operation time and radiation exposure compared with the conventional CT-guided radiofrequency ablation.

## Introduction

Osteoid osteoma (OO) is a common benign bone tumor accounting for approximately 5 % of all bone tumors (1). Adolescents and young adults are the most common groups diagnosed with this tumor. Clinically there is severe local pain that typically worsens at night. Though some patients have pain relief, most of them require surgical intervention for faster pain relief and to reduce the recurrence rate.

Open surgery involves excessive removal of large portions of cortical bone, resulting in post-operative complications. As a thermal tissue destruction technique, radiofrequency ablation (RFA) has been applied to the treatment of OO with satisfactory results (2). Currently, percutaneous RFA with CT guidance for the treatment of OO has been widely applied (3). However, since OO is common among children, the multiple instances of radiation exposure in the CT-guided RFA process might put them at risk for future radiation-related complications (4).

Three-dimensional imaging system (O-arm) has been used for a wide variety of cases for its three-dimensional intra-operative imaging property (5). Besides, minimally invasive treatment technology represented by surgical robots has also become one of the main directions of surgical development (6). With precise, minimally invasive, and efficient properties, robot-assisted surgery has shown great potential in clinical applications (7).

The purpose of this study is to compare the clinical effects of robot-assisted RFA with O-arm navigation with the conventional CT-guided RFA.

## Materials and Methods

### Patients

The retrospective analysis was approved by the ethics committee of our hospital. Patients who underwent robot-assisted RFA or CT-guided RFA treatment for OO between 2005 and 2018 were included. The inclusion criteria for this study were as follows: (1) Intra-operative pathological specimens were confirmed to be OO; (2) all patients were diagnosed with OO based on clinical findings and radiology studies; (3) following time >12 months; and (4) no prior surgical treatment. Finally, a total of 62 patients with pain as the most distinct symptom were involved in this study (Robot-RFA group, *n* = 24; CT-RFA group, *n* = 38). The mean follow-up time was 22.00 ± 7.11 months (range, 12–36) for the Robot-RFA group and 24.10 ± 6.40 months (range, 12–39) for the CT-RFA group. The general information of the two groups is displayed in Table 1.

**Table 1:** General information of the two groups.

**Characteristics**	**Robot-RFA (*n* = 24)**	**CT-RFA (*n* = 38)**	***p*-value**
Age (years)	13.83 ± 7.23	16.58 ± 8.20	0.18
Gender (male/female)	11/13	17/21	0.60
Lesion localization			0.39
Femur	11	21	
Tibia	6	12	
Fibula	3	2	
Humerus	2	3	
iliac	2	0	
Lesion size (mm)	5.30 ± 1.23	5.14 ± 1.00	0.59
Preoperative VAS	7.17 ± 0.92	7.05 ± 0.98	0.63
Follow-up time (months)	22.00 ± 7.11	24.10 ± 6.40	0.23

*Robot-RFA, Robot-assisted, O-arm-navigated radiofrequency ablation; CT-RFA, CT-guided radiofrequency ablation; VAS, visual analog scale*.

### Surgical Intervention

**Figure 1:**
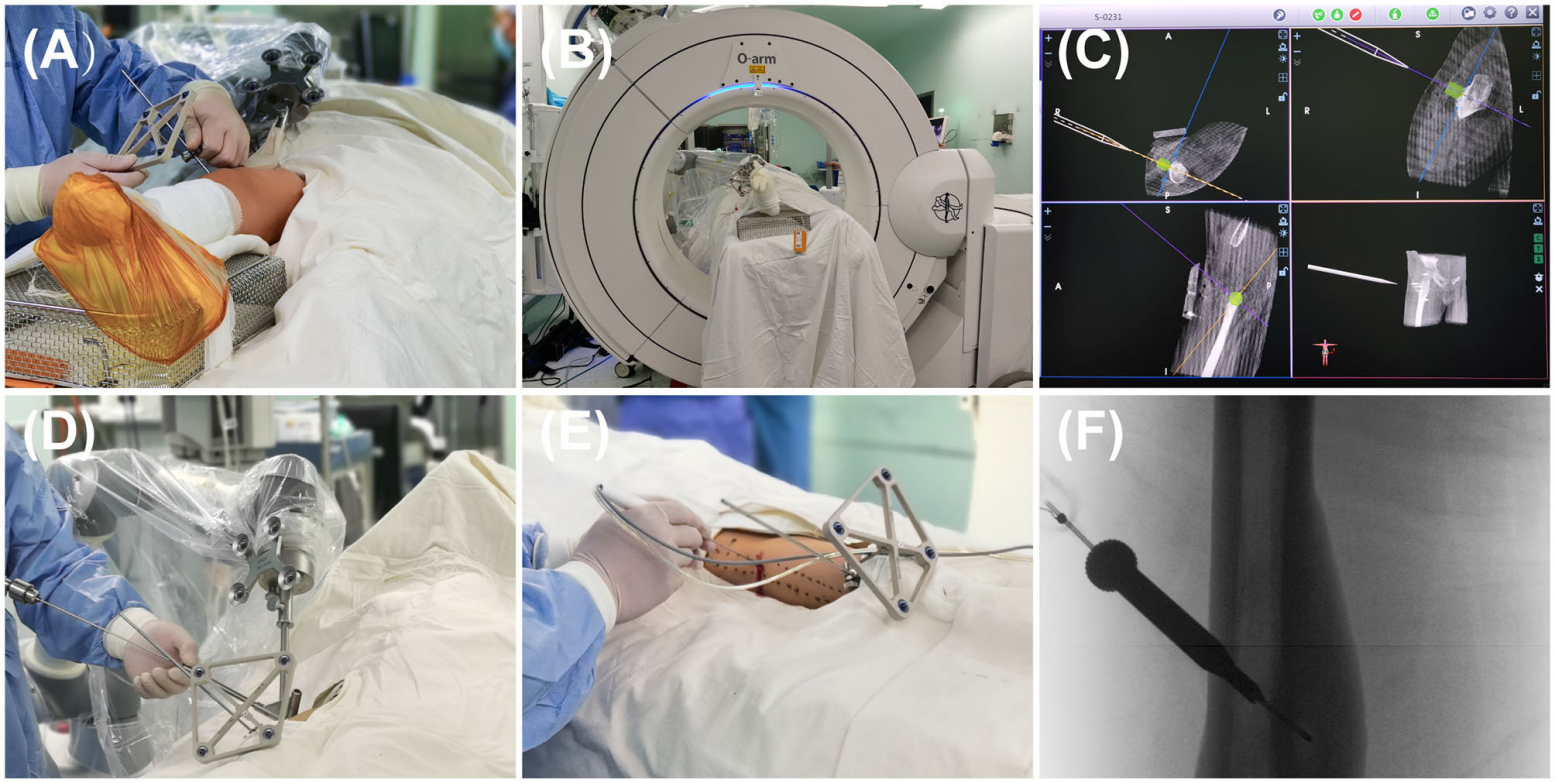
The process of robot-assisted, O-arm-navigated radiofrequency ablation (RFA) of osteoid osteoma (OO). **(A)** Tracer installation for the real-time capture of patient's spatial location. **(B)** Collecting the preoperative three-dimensional (3D) radiographs data and uploading it to the robot system. **(C)** 3D reconstructions and surgical path planning in the robot system. **(D)** The movement of robot's arm and director following the planned path, followed by the penetration of K-wire into the nidus with the assistance of robot's director. **(E)** The placement of RF needle into the nidus following the path of K-wire. **(F)** The radiographs of the RF needle localization.

In the Robot-RFA group, patients were placed in a prone or supine position on a carbon-fiber bed under general anesthesia. Before the surgery, the skin was sterilized based on the standard process, and machines including an O-arm (Medtronic, USA) and a robot (TINAVI Medical Technologies, China) were wrapped with sterile sleeves. According to the anatomical location of the lesion, the optical tracer was fixed at a proper position on the patient (Figure 1A). The patient was then scanned by the O-arm to locate the lesion (Figure 1B). The obtained three-dimensional (3D) images were then created and uploaded to the robot imaging system. The needle path was then designed according to the 3D images.

Next, the surgeon simulated the movement of the manipulator's arm in the robot operating system based on the designed path and confirmed that the angle and direction were correct (Figure 1C). According to the designed path, a very small skin incision of about 3 mm was made to avoid damage of soft tissue caused by tissue entanglement when the K-wire was inserted, and then a K-wire penetrated through the guide sleeve of the director into the nidus (Figure 1D). The position of the needle was then adjusted according to the repeat O-arm scan images. An RF needle (RITA Angiodynamics Inc., USA) was then advanced following the path of the K-wire (Figure 1E). When the needle came into the lesion as planned, a repeat scan was performed to confirm the location of the RF needle (Figure 1F), after which the RFA was performed. At last, the incision was sutured with nylon sutures, and the patient's skin was examined for burns or other superficial complications.

In the CT-guided percutaneous excision, the localization of the nidus was first determined by the preoperative 3D CT scan at a thickness of 1 mm, and then a K-wire was advanced to the nidus according to the experience of the operator. The K-wire could be adjusted several times to achieve an appropriate angle and direction in order to place the tip of K-wire in the center of the nidus under the guidance of CT sweep (15, 16). All lesions in the two groups were treated with RFA at 90° for 6–8 min with gradual increase of heat.

### Data Collection and Assessment

Intra-operative data included operation time, dose-length product (DLP), and mean K-wire adjustment times. Operative time was defined as the time from skin sterilization to skin suture. DLP was defined as the total absorbed dose of radiation exposure, which is useful and easily acquired for comparing exam doses and measured in milligray-centimeter (mGy-cm^2^) (17). Mean K-wire adjustment times represent the accuracy of surgery.

Post-operative data included primary technical success rate, visual analog scale (VAS), lesion size, post-operative complications, and radiological outcomes. VAS was applied to assess the pain relief before and after surgery at 24 h. The lesion size was measured according to the radiological images during the follow-up time. Nerve palsy and infection were recorded as the complications. The cortical changes were also noted.

### Statistical Analysis

The collected data were analyzed by the SPSS 25.0 software (SPSS Inc., Chicago, USA). Continuous variables are expressed as Mean ± standard deviation (SD) and categorical variables are expressed as the frequency with percentages. Student *t*-test was performed to compare the data of the two independent groups. A Chi-square test was performed to analyze the categorical variables from the independent groups. Fisher's exact test was performed if an expected number was <5. A *p*-value of < 0.05 was considered statistically significant.

## Results

### General Information of the Two Groups

Data of 62 patients with OO (24 patients received the robot-assisted RFA and 38 patients received the CT-guided RFA) were collected in this study. The mean age was 13.83 ± 7.23 years in the Robot-RFA group and 16.58 ± 8.20 years in the CT-RFA group. The Robot-RFA group comprised 11 males and 13 females, and the CT-RFA group comprised 17 males and 21 females.

Most of the lesions were located at the femur and tibia, while the other localizations included the fibula, the humerus, the iliac, and the calcaneus. The pre-operative mean lesion size was 5.30 ± 1.23 mm in the Robot-RFA group and 5.14 ± 1.00 mm in the CT-RFA group. The mean pre-operative VAS was 7.17 ± 0.92 in the Robot-RFA group and 7.05 ± 0.98 in the CT-RFA group. All patients in the two groups were followed up for at least 12 months.

In conclusion, there was no statistically significant difference in the age, gender, localization of lesion, pre-operative lesion size, pre-operative VAS score, and follow-up time between the two groups (Table 1).

### Intra-Operative and Post-operative Measurements

The operation time was 40.29 ± 9.05 min in the Robot-RFA group, while it was 58.18 ± 12.47 min in the CT-RFA group. The operation time was shorter in the Robot-RFA group (*p* < 0.05). Meanwhile, less radiation exposure was observed in the Robot-RFA group. The DLP was 436.25 ± 327.66 mGy-cm^2^ in the Robot-RFA group and 776.05 ± 474.58 mGy-cm^2^ in the CT-RFA group (*p* < 0.05). In addition, the K-wire adjustment times was were only 0.21 ± 0.41 times in the Robot-RFA group, while 1.45 ± 0.80 times in the CT-RFA group (*p* < 0.05).

The primary technical success rate was 95.8% (23/24) in the Robot-RFA group and 92.1% (35/38) in the CT-RFA group. Patients with pain recurrence received repeat-RFA and had a secondary success rate of 100%. The VAS score at 24 h after surgery was 1.29 ± 1.12 in the Robot-RFA group and 1.18 ± 1.18 in the CT-RFA group. The mean lesion size of OO was 3.40 ± 1.01 mm in the Robot-RFA group and 2.68 ± 0.75 mm in the CT-RFA group at 6 months. However, the primary technical success rate, VAS score at 24 h, and lesion size at 6 months after surgery showed no statistical significance between the two groups (*p* > 0.05). No complications associated with the procedure were reported in the two groups during the follow-up period.

In conclusion, there was a statistically significant difference in the operation time, dose of radiation exposure, and mean K-wire adjustment times between the two groups. No significant difference was observed in the primary technical success rate, VAS score at 24 h, and lesion size at 6 months after surgery (Table 2).

**Table 2:** Comparative analysis of postoperative outcomes between the two groups.

**Variables**	**Robot-RFA** **(*n* = 24)**	**CT-RFA** **(*n* = 38)**	***p* value**
Operation time (min)	40.29 ± 9.05	58.18 ± 12.47	<0 0.01^*^
DLP (mGy-cm^2^)	436.25 ± 327.66	776.05 ± 474.58	<0 0.01^*^
K-wire adjustment times	0.21 ± 0.41	1.45 ± 0.80	<0 0.01^*^
Primary technical success rate	95.8 (23/24)	92.1% (35/38)	0.56
VAS (24 h)	1.29 ± 1.12	1.18 ± 1.18	0.52
Lesion size (6th month, mm)	3.40 ± 1.01	2.68 ± 0.75	0.43

*Robot-RFA, Robot-assisted, O-arm-navigated radiofrequency ablation; CT-RFA, CT-guided radiofrequency ablation; DLP, dose-length product; VAS, visual analog scale*.

* *Statistically significant*.

## Discussion

Radiofrequency ablation with CT-guided navigation represented the most popular minimally invasive technique for the treatment of OO, and has been performed for several years. Much attention has also been paid to robot-assisted surgery and 3D navigation as novel techniques in clinical treatment. In this retrospective study, we compared the surgical effect of robot-assisted RFA with O-arm navigation with the conventional CT-guided RFA in the treatment of OO. Though the primary technical success rate, post-operative VAS score, and post-operative lesion size showed no significant difference between the two groups, the operation time, dose of radiation exposure, and accuracy of surgery showed that robot-assisted RFA is superior to conventional CT-guided RFA.

The most recommended and effective treatment for OO is surgical excision, which could completely remove the lesion and surrounding bone (18). However, the excessive removal of large portions of cortical bone in open surgery destroys the stability of the bone, resulting in a higher incidence of avascular necrosis and post-operative fractures (19). High post-operative complications have also taken place in open excision surgery for OO because of the inaccurate localization of the nidus (20). Over the decades, minimally invasive surgery represented by the CT-guided surgery and percutaneous RFA has become the trend worldwide in the treatment of OO (21). Many studies have demonstrated that CT-guided RFA is an acceptable strategy with minimal damage and fewer complications in the treatment of OO compared with open excision surgery (12). However, with the increasing usage of such CT guidance, radiation exposure has become a main concern, especially for young children, who are most susceptible to this disease. Some studies also highlighted the importance of minimizing radiation exposure in the adolescent population (22, 23). Nowadays, more attention is being paid to robot-assisted surgery and 3D navigation for their great potential in clinical application (24). Compared with the CT scan, O-arm can decrease the radiation exposure to both surgical staff and patients (25, 26). Kadar et al. (8) reported their experience in 52 O-arm-guided RFA procedures and measured a mean DLP of 544.7 mGy-cm^2^, which was significantly lower than previously reported data regarding DLP of ablations with conventional CT guidance. Cheng et al. (9) have compared the use of an O-arm navigated system with conventional CT guidance for OO ablation in a case-control study, and a significant difference in DLP also existed between the two groups. Tsalafoutas et al. (10) performed CT-guided RFA in 14 cases with a mean DLP of 1976 mGy-cm^2^. Renhitz et al. (11) performed CT-guided RFA in 102 cases with a mean DLP of 751.55 mGy-cm^2^. All the CT-guided RFA showed a much higher radiation exposure than that of the reported O-arm navigated RFA (Table 3). Most of these reported studies showed a low recurrence and complication rate with variations (12, 13), which may be explained by the difference in localization of the lesion and the surgeon's experience in operation. Rimondi et al. (14) retrospectively studied 557 patients who received CT-guided RFA, and found that the clinical results were greatly improved *via* regulating the temperature and time of RFA. In recent years, robot-assisted surgery is also an evolving technology that has been given more attention in orthopedics. Due to the advantages of precise operation and greater reduction of radiation exposure, robot-assisted surgery in orthopedics has been performed in our institution, including spinal fractures, limb fractures, pelvic fractures, and OO, which have received excellent clinical results as reported (27).

**Table 3:** Summary of some reported studies on using of O-arm or CT-guided RFA for the treatment of OO.

**Authors**	**Patients**	**Type of guidance**	**Mean DLP (mGy-cm2)**	**Recurrence**	**Complications**	**References**
Wang et al. (this study)	24	O-arm	436.25	1	0	
Kadar et al.	52	O-arm	544.7	3	1	(8)
Cheng et al.	23	O-arm	446.6	2	0	(9)
Cheng et al.	36	CT	1058.8	3	1	(9)
Tsalafoutas et al.	14	CT	1976	/	/	(10)
Renhitz et al.	102	CT	751.55	1	0	(11)
Cuesta et al.	200	CT	/	4	3	(12)
Lassalle et al.	126	CT	/	4	4	(13)
Rimondi et al.	557	CT	/	24	5	(14)

*RFA, radiofrequency ablation; CT, computer tomography; DLP, dose-length product; OO, osteoid osteoma*.

**Figure 2:**
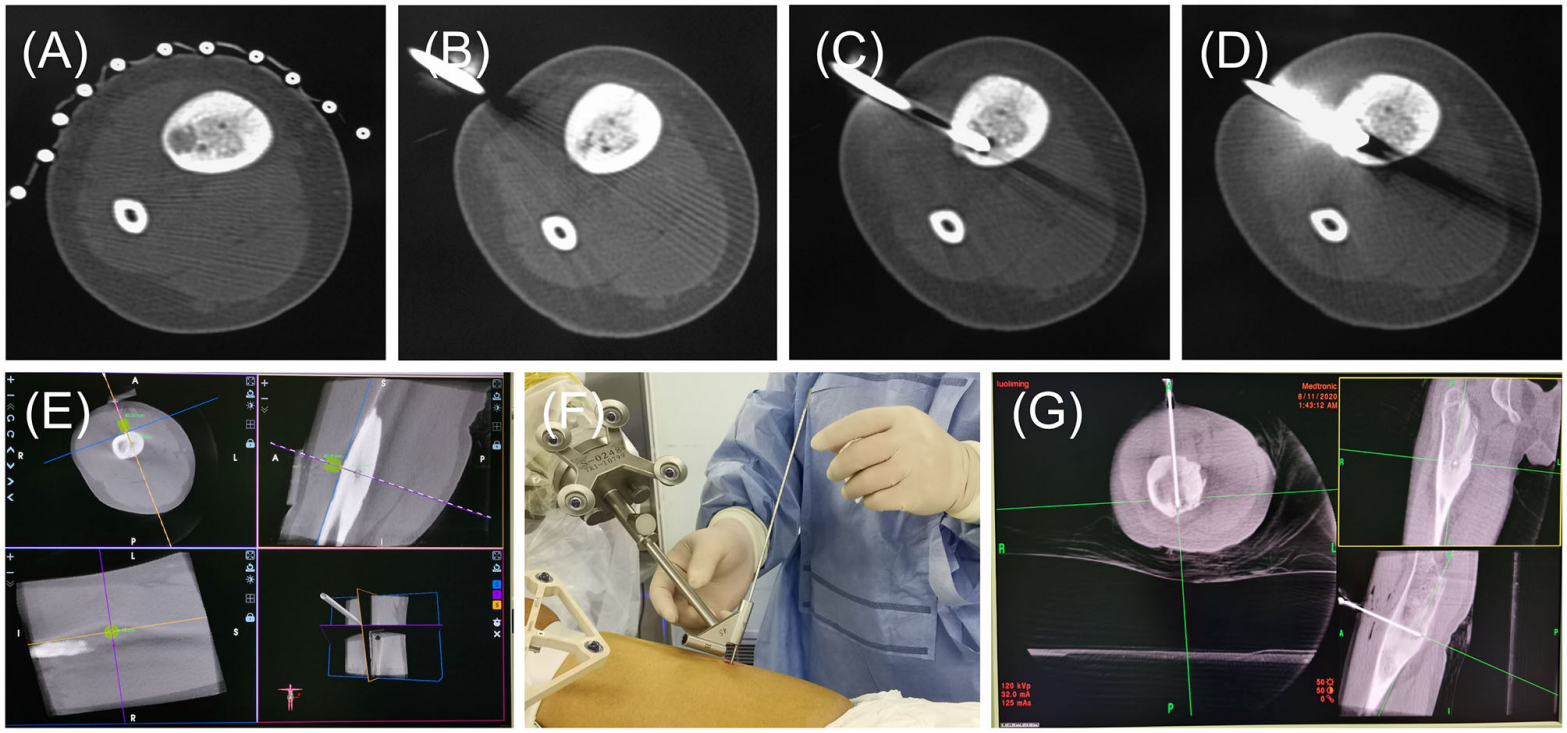
The radiographs of general operative procedures of the CT-RFA group and Robot-RFA group. **(A)** Pre-operative CT scan. **(B–D)** Radiographs of CT scan for the K-wire localization and confirmation. **(E)** Guide pin path planning. **(F)** K-wire insertion follows the path of sleeve of the robot. **(G)** Location confirmation of the K-wire.

**Figure 3:**
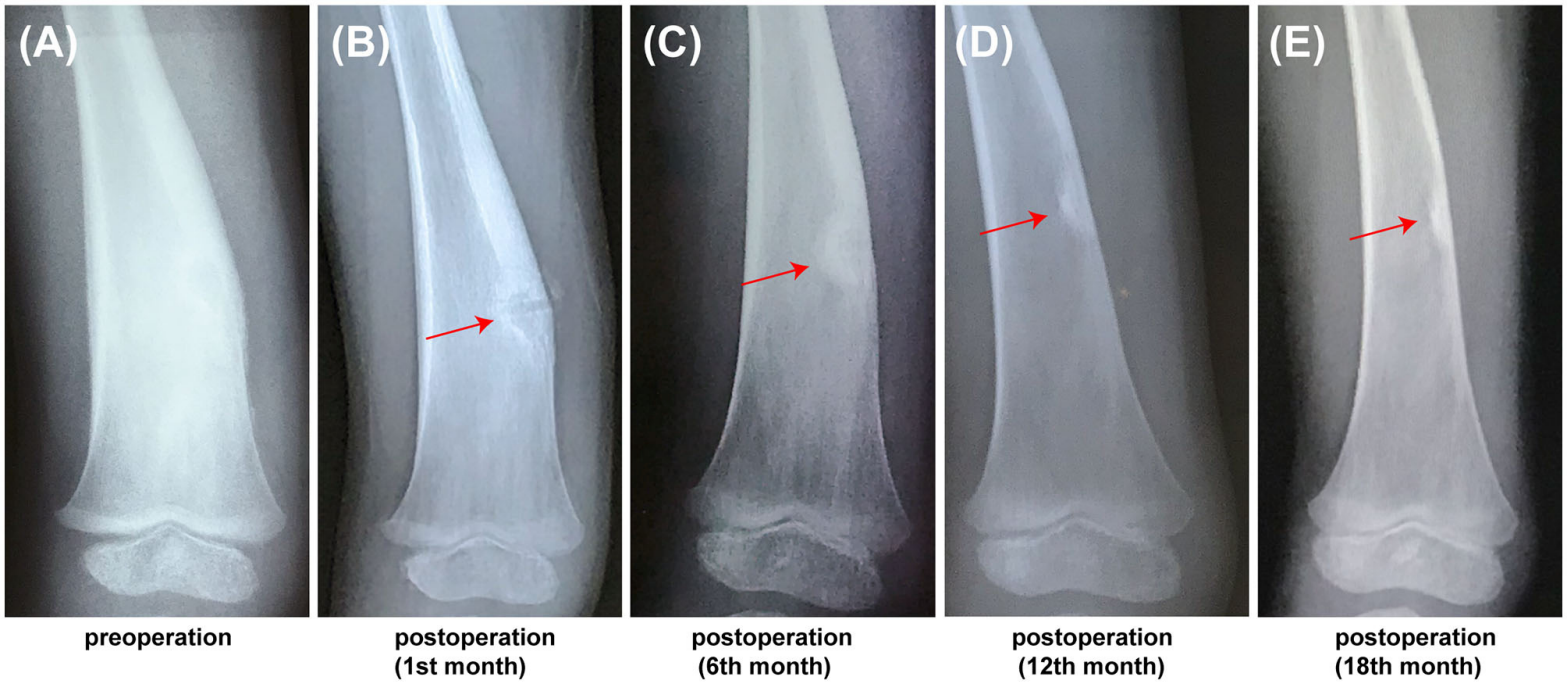
The follow-up radiographs of a 3-year-old child confirmed with OO using the robot-assisted surgery. **(A)** Pre-operative X-ray image showed showing the thickening of cortex. **(B)** PostoperativePost-operative X-ray image at 1st month, **(C)** Post-operative X-ray image at 6th month. **(D)** Post-operative X-ray image at 12th month. **(E)** Post-operative X-ray image at 18th month (red arrow, the nidus).

**Figure 4:**
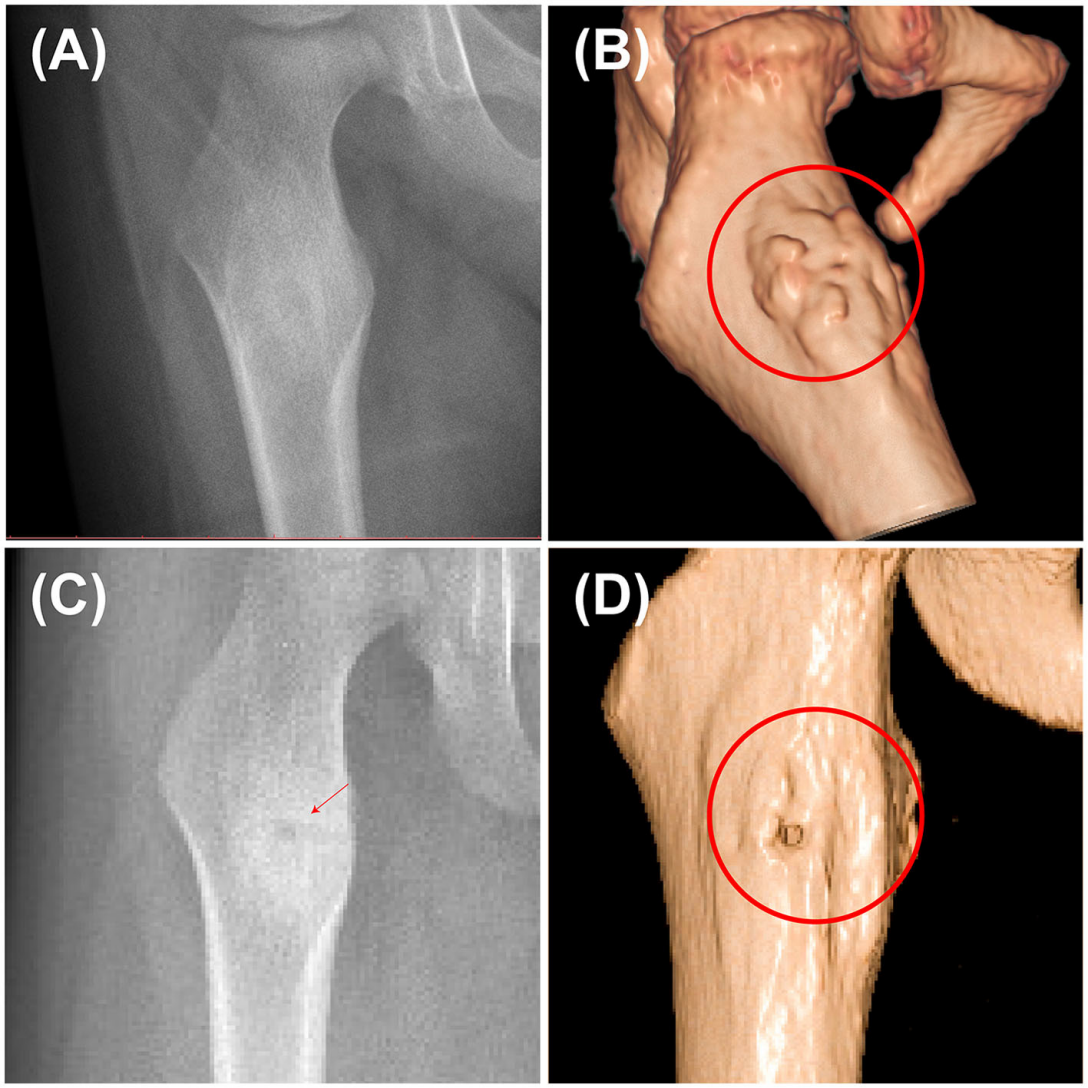
Pre-operative and post-operative radiographs of a 3-year-old child confirmed with OO using the robot-assisted surgery. **(A)** Pre-operative X-ray image of the nidus. **(B)** Pre-operative 3D reconstruction image showing the thickening of the nidus. **(C)** Post-operative X-ray image at 3rd month. **(D)** Post-operative 3D reconstruction at 3rd month showing that the nidus greatly shrank (red arrow and cycle, the nidus).

**Figure 5:**
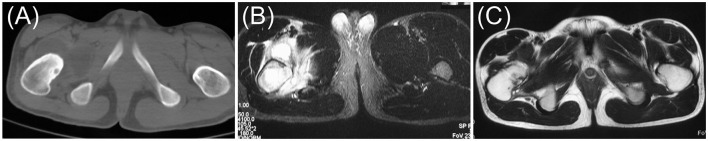
Pre-operative and post-operative radiographs of a 17-year-old male confirmed with OO using the robot-assisted surgery. **(A)** Pre-operative axial CT image. **(B)** Pre-operative axial MR image. **(C)** Post-operative axial MR image at 12th month (red arrow, the nidus).

In this study, the accuracy and safety of robot-assisted RFA with O-arm navigation in the treatment of OO have been confirmed. The operation time was shorter in the Robot-RFA group compared with the CT-guided RFA group, which was mainly due to the assistance of the robot system. In the CT-guided RFA group, the operator could only insert the K-wire and sleeve based on the pre-operative CT scan (Figure 2A), which could result in the requirement to adjust the path several times during the operation with the navigation of CT scan (Figures 2B–D). The time of navigation will be much more for less experienced operators. However, with the assistance of robot system in the robot-assisted group to design the needle path according to the 3D images (Figure 2E), multiple adjustments of K-wire were unnecessary, and the K-wire could be inserted to a perfect region (Figures 2F,G). Compared with CT-guided RFA, the robot-assisted surgery was more efficient and accurate in the treatment of OO, which could further reduce the dose of radiation exposure. As a result, mean radiation exposure in 24 procedures of robot-assisted RFA was 436.25 mGycms^2^, which was much lower than conventional CT-guided RFA and also lower than the data reported using O-arm guided RFA without robot assistance (8). Meanwhile, this technique could provide excellent clinical results with significant pain relief, and no complications were observed as in conventional CT-guided RFA. Shown in Figure 3, is a representative case of a 3-year-old child confirmed with OO and treated with robot-assisted RFA with O-arm navigation. Pain relief was observed to a great extent after operation, and radiographs showed that the nidus gradually shrank and achieved advanced bone healing during the follow-up period (Figures 3B–E). A recent case who received robot-assisted RFA with O-arm navigation is shown in Figure 4. The pre-operative radiographs and 3D reconstruction showed a small nidus with cortical thickening (Figures 4A,B). Specifically, at a follow-up of 3 months, excellent pain relief was achieved and the size decreased markedly according to the post-operative X-ray and 3D reconstruction (Figures 4C,D). Another case confirmed with OO showed increased contrast enhancement of the nidus and the surrounding tissue edema in the MR image (Figures 5A,B). The accompanying tissue edema was greatly decreased (Figure 5C) after treatment with robot-assisted surgery on follow-up examination.

Finally, a few limitations existed in this study. Firstly, the investigation was performed at one single center with a small group of patients. Secondly, the patients in the two groups were retrospectively collected during different time intervals. Third, it is a retrospective analysis without similar lesion locations in these patients. Finally, a cost analysis was not performed, although the cost was higher when the robot surgery was applied.

## Conclusions

This study favors the results that robot-assisted RFA with O-arm navigation is a more accurate and safer technique with less operation time and radiation exposure compared with the conventional CT-guided RFA. The K-wire adjustment times that represent the accuracy of surgery in this present investigation demonstrated that robot-assisted RFA is a much more efficient and safe technique. To our knowledge, we also believe that this technique has a great potential to further increase the accuracy of surgery and also reduce the expenses of patients by increasing the operator's proficiency in this technique.

## Data Availability Statement

The original contributions presented in the study are included in the article/supplementary material, further inquiries can be directed to the corresponding author.

## Ethics Statement

The study was approved by the Ethics Committee of the Shanghai Tenth People's Hospital, No. SHSY-IEC-4.1/21-47/01. All the methods were performed in accordance with the relevant guidelines and regulations, such as the Declaration of Helsinki. Due to the retrospective design of the study, the written informed consent was waived by the Ethics Committee of Shanghai Tenth People's Hospital and all data were anonymized.

## Author Contributions

T-LW designed the study and wrote the article. All authors contributed to the article and approved the submitted version.

## Funding

This research was funded by the Science and Technology Commission of Shanghai Municipality, grant number: 21ZR1450000.

## Conflict of Interest

The authors declare that the research was conducted in the absence of any commercial or financial relationships that could be construed as a potential conflict of interest.

## Publisher's Note

All claims expressed in this article are solely those of the authors and do not necessarily represent those of their affiliated organizations, or those of the publisher, the editors and the reviewers. Any product that may be evaluated in this article, or claim that may be made by its manufacturer, is not guaranteed or endorsed by the publisher.
